# Salvage chemotherapy for adults with relapsed or refractory lymphoma in Malawi

**DOI:** 10.1186/s13027-017-0156-3

**Published:** 2017-08-09

**Authors:** Bongani Kaimila, Toon van der Gronde, Christopher Stanley, Edwards Kasonkanji, Maria Chikasema, Blessings Tewete, Paula Fox, Satish Gopal

**Affiliations:** 1 UNC Project-Malawi, Private Bag A-104, Lilongwe, Malawi; 2 University of North Carolina, Chapel Hill, USA; 3 University of Malawi College of Medicine, Blantyre, Malawi

**Keywords:** Non-Hodgkin lymphoma, Hodgkin lymphoma, Sub-Saharan Africa, Chemotherapy, HIV

## Abstract

**Background:**

Lymphoma is highly associated with HIV in sub-Saharan Africa (SSA), which contributes to worse outcomes relative to resource-rich settings, and frequent failure of first-line chemotherapy. However, there are no second-line treatment descriptions for adults with relapsed or refractory lymphoma (RRL) in SSA.

**Methods:**

We describe HIV+ and HIV- patients with RRL receiving salvage chemotherapy in Malawi. Patients were prospectively treated at a national teaching hospital in Lilongwe, with the modified EPIC regimen (etoposide, prednisolone, ifosfamide, cisplatin) between June 2013 and May 2016, after failing prior first-line chemotherapy.

**Results:**

Among 21 patients (18 relapsed, 3 refractory), median age was 40 years (range 16–78), 12 (57%) were male. Thirteen patients (62%) were HIV+, of whom 12 (92%) were on antiretroviral therapy (ART) at initiation of salvage chemotherapy, with median CD4 cell count 139 cells/μL (range 12–529) and 11 (85%) with suppressed HIV RNA. Median number of EPIC cycles was 3 (range 1–6), and the commonest toxicity was grade 3/4 neutropenia in 19 patients (90%). Fifteen patients responded (3 complete, 12 partial, overall response rate 71%), but durations were brief. Median overall survival was 4.5 months [95% confidence interval (CI) 2.4–5.6]. However, three patients, all HIV+, experienced sustained remissions. Tolerability, response, and survival did not differ by HIV status.

**Conclusions:**

The appropriateness and cost-effectiveness of this approach in severely resource-limited environments is uncertain, and multifaceted efforts to improve first-line lymphoma treatment should be emphasized, to reduce frequency with which patients require salvage chemotherapy.

**Trial registration:**

NCT02835911. Registered 19 January 2016.

## Introduction

Lymphoma incidence has increased in sub-Saharan Africa (SSA) due to human immunodeficiency virus (HIV) and population aging [1]. While many patients can be cured, long-term survival remains suboptimal due to typically advanced disease, limited chemotherapy formularies and treatment intensity, poor supportive care without hematopoietic growth factors, limited access to targeted agents, and scarce radiotherapy [2–4]. To illustrate, in Malawi, we have reported 45% 1-year overall survival (OS) for aggressive non-Hodgkin lymphoma (NHL) and 75% for classical Hodgkin lymphoma (HL) [5, 6]. As a result, relapsed or refractory lymphoma (RRL) is common, for which there is no standard treatment. To our knowledge, there are no published descriptions of salvage treatment for RRL among adults in SSA. We therefore report our experience with the first prospectively treated cohort of patients in Malawi receiving second-line chemotherapy.

## Methods

Patients were treated at Kamuzu Central Hospital (KCH) in Lilongwe, Malawi’s capital. KCH is a national teaching hospital which provides cancer care to half the country’s 17 million people. At initial diagnosis of lymphoma, all patients were invited to participate in the KCH Lymphoma Study longitudinal cohort, for which procedures have been described in detail [5–7]. The study was conducted in accordance with the Helsinki declaration after approval by the University of North Carolina institutional review board and Malawi National Health Sciences Review Committee. All diagnoses were pathologically confirmed using biopsies supported by immunohistochemistry (IHC) and weekly real-time telepathology consultation involving 2–4 Malawian and United States pathologists [8, 9]. First-line treatment was CHOP (cyclophosphamide, doxorubicin, vincristine, prednisone) for aggressive NHL and ABVD (doxorubicin, bleomycin, vinblastine, dacarbazine) for HL [5, 6]. In this paper, we restricted analyses to adults >16 years with RRL treated with the modified EPIC (etoposide, prednisone, ifosfamide, cisplatin) regimen between June 2013 and May 2016 after prior first-line chemotherapy.

We were motivated to develop a salvage chemotherapy program as a result of the frequent clinical need to palliate RRL patients with significant tumor bulk and cancer-related symptoms in Lilongwe, often with good performance status. We chose the modified EPIC regimen due to lack of cross-resistance with first-line chemotherapy, drug availability in our environment, and prior description of its use in high-income countries in the ambulatory setting without hematopoietic growth factors and with manageable toxicities [10]. Moreover, the regimen bears similarity to the more commonly used ICE (ifosfamide, carboplatin, etoposide) salvage regimen in resource-rich countries, without requiring high-dose 24-h infusion of ifosfamide which is impractical in our setting. Notably, targeted agents including rituximab and brentuximab vedotin are not available in the Malawi public sector for first- or second-line use, nor is radiotherapy.

We administered modified EPIC in Lilongwe as follows: etoposide intravenously 100 mg/m^2^ days 1–4, ifosfamide intravenously 1000 mg/m^2^ days 1–4; mesna intravenously at 60% of the ifosfamide dose days 1–4; prednisolone orally 60 mg/m^2^ days 1–5; cisplatin intravenously 75 mg/m^2^ day 15. The regimen was administered with 28-day cycles. Complete blood count, renal, and hepatic function were assessed before chemotherapy administration on days 1 and 15 of each cycle. If absolute neutrophil count (ANC) was less than 0.75 × 10^3^ cells/μL on scheduled day 1, treatment was delayed for one week until recovery. If ANC was less than 0.75 × 10^3^ cells/μL or serum creatinine was increased by more than 25% from baseline on scheduled day 15, cisplatin was omitted. Patients could receive up to six cycles if responding without severe adverse events related to therapy. For anti-infective prophylaxis during treatment, all patients received ciprofloxacin and HIV-positive patients additionally received fluconazole. HIV-infected individuals were maintained on antiretroviral therapy (ART) and cotrimoxazole as per national HIV treatment guidelines. Tumor response was evaluated using physical exam, chest x-ray, and abdominal ultrasound. Complete response (CR) was defined as resolution of all assessable tumor sites. Partial response (PR) was defined as >50% reduction of the assessable baseline tumor burden.

## Results

Between June 2013 and May 2016, 21 patients were treated with modified EPIC in Lilongwe (Table 1). Median age was 40 years (range 16–78). Thirteen patients (62%) were HIV-positive, of whom 12 (92%) were on ART at initiation of salvage chemotherapy, with median CD4 cell count of 139 cells/μL (range 12–529) and 11 (85%) with suppressed HIV RNA. Aggressive B-cell NHLs (including diffuse large B-cell lymphoma, Burkitt lymphoma, plasmablastic lymphoma, and aggressive B-cell NHL not otherwise specified) were most frequently represented (18, 86%), with most patients having relapsed disease after prior locally adjudicated remission (18, 86%), although median time to relapse after completion of first-line chemotherapy was short (3.0 months, range 0.7–19.4). Most patients had localized disease (12, 57%), significant tumor bulk (median 6.5 cm, range 3–22), performance status ≤2 (18, 86%), and preserved bone marrow and kidney function prior to EPIC initiation.

**Table 1:** Baseline characteristics of patients with relapsed/refractory lymphoma initiating salvage chemotherapy in Lilongwe

	Total (*n* = 21)	HIV+ (*n* = 13)	HIV- (*n* = 8)
Age, years	40 (16–78)	49 (16–63)	22 (18–78)
Male, n (%)	12 (57%)	8 (62%)	4 (50%)
Histologic diagnosis, n (%)			
Diffuse large B-cell lymphoma	6 (29%)	3 (23%)	3 (38%)
Burkitt lymphoma	2 (10%)	―	2 (25%)
Plasmablastic lymphoma	2 (10%)	2 (15%)	―
Aggressive B-cell NHL NOS	8 (38%)	8 (62%)	―
Classical Hodgkin lymphoma	3 (14%)	―	3 (38%)
Refractory, n (%)	3 (14%)	1 (8%)	2 (25%)
Relapsed, n (%)	18 (86%)	12 (92%)	6 (75%)
Time to relapse from first-line chemotherapy completion, months	3.0 (0.7–19.4)	3.0 (0.7–19.4)	3.6 (0.8–8.1)
Localized disease, n (%)	12 (57%)	10 (77%)	2 (25%)
Largest lymph node mass, cm	6.5 (3–22)	16 (5–35)	11 (2–18)
Performance status ≤2, n (%)	18 (86%)	12 (92%)	6 (75%)
White blood cells, 10^3^/μL	4.6 (2.7–18.6)	4.4 (3.3–8.9)	4.9 (2.7–18.6)
Absolute neutrophil count, 10^3^/μL	2.9 (0.9–14.3)	2.4 (0.9–6.3)	3.0 (1.3–14.3)
Hemoglobin, g/dL	11.2 (6.0–14.7)	12.2 (9.2–14.7)	10.3 (6.0–14.0)
Platelets, 10^3^/μL	332 (56–767)	312 (204–655)	353 (56–767)
Serum creatinine, mg/dL	0.7 (0.3–2.9)	0.8 (0.4–2.9)	0.6 (0.3–1.0)
eGFR <60 mL/min/1.73m^2^, n (%)	1 (5%)	―	1 (13%)
CD4 count if HIV+, cells/μL	―	139 (12–529)	―
HIV RNA <400 copies/mL if HIV+, n (%)	―	11 (85%)	―

Values indicate median (range) unless otherwise specified

*NHL* non-Hodgkin lymphoma, *NOS* not otherwise specified, *eGFR* estimated glomerular filtration rate

Treatment course and toxicities for patients receiving EPIC are shown in Table 2. The median number of cycles was 3 (range 1–6), with disease progression being the primary reason for therapy discontinuation. Number of cycles, cumulative dose, and dose intensity were overall similar between HIV-positive and HIV-negative patients. Grade 3/4 neutropenia occurred in 19 patients (90%), and was responsible for only two patients (10%) receiving at least 50% of planned day 15 cisplatin doses. Grade 3/4 anemia and thrombocytopenia occurred in 10 (48%) and three (14%) patients, respectively. Grade 3/4 renal dysfunction occurred in only one patient, and of eight patients with grade 3/4 other non-hematologic toxicities, these included pain (4), peripheral neuropathy (3), and nausea/vomiting (1).

**Table 2:** Treatment course and toxicities among patients with relapsed/refractory lymphoma receiving modified EPIC salvage chemotherapy in Lilongwe

	Total (*n* = 21)	HIV+ (*n* = 13)	HIV- (*n* = 8)
Cycles per patient	3 (1–6)	3 (1–6)	3.5 (1–6)
Days between cycles	28 (25–45)	28 (25–42)	28 (25–45)
Etoposide dose per cycle, mg/m^2^	100 (48–104)	99 (51–104)	100 (48–102)
Ifosfamide dose per cycle, mg/m^2^	1000 (482–1042)	993 (511–1042)	1000 (482–1019)
Received >50% of day 15 cisplatin doses, n (%)	2 (10%)	1 (8%)	1 (13%)
Received <4 cycles, n (%)	12 (57%)	8 (62%)	4 (50%)
Progression	11	7	4
Social	1	1	―
Any grade 3/4 neutropenia, n (%)	19 (90%)	11 (86%)	8 (100%)
Any grade 3/4 anemia, n (%)	10 (48%)	4 (31%)	6 (75%)
Any grade 3/4 thrombocytopenia, n (%)	3 (14%)	1 (8%)	2 (25%)
Any grade 3/4 renal dysfunction, n (%)	1 (5%)	1 (8%)	―
Any grade 3/4 other non-hematologic toxicity, n (%)	8 (38%)	4 (31%)	4 (50%)

Values indicate median (range) unless otherwise specified

**Fig. 1:**
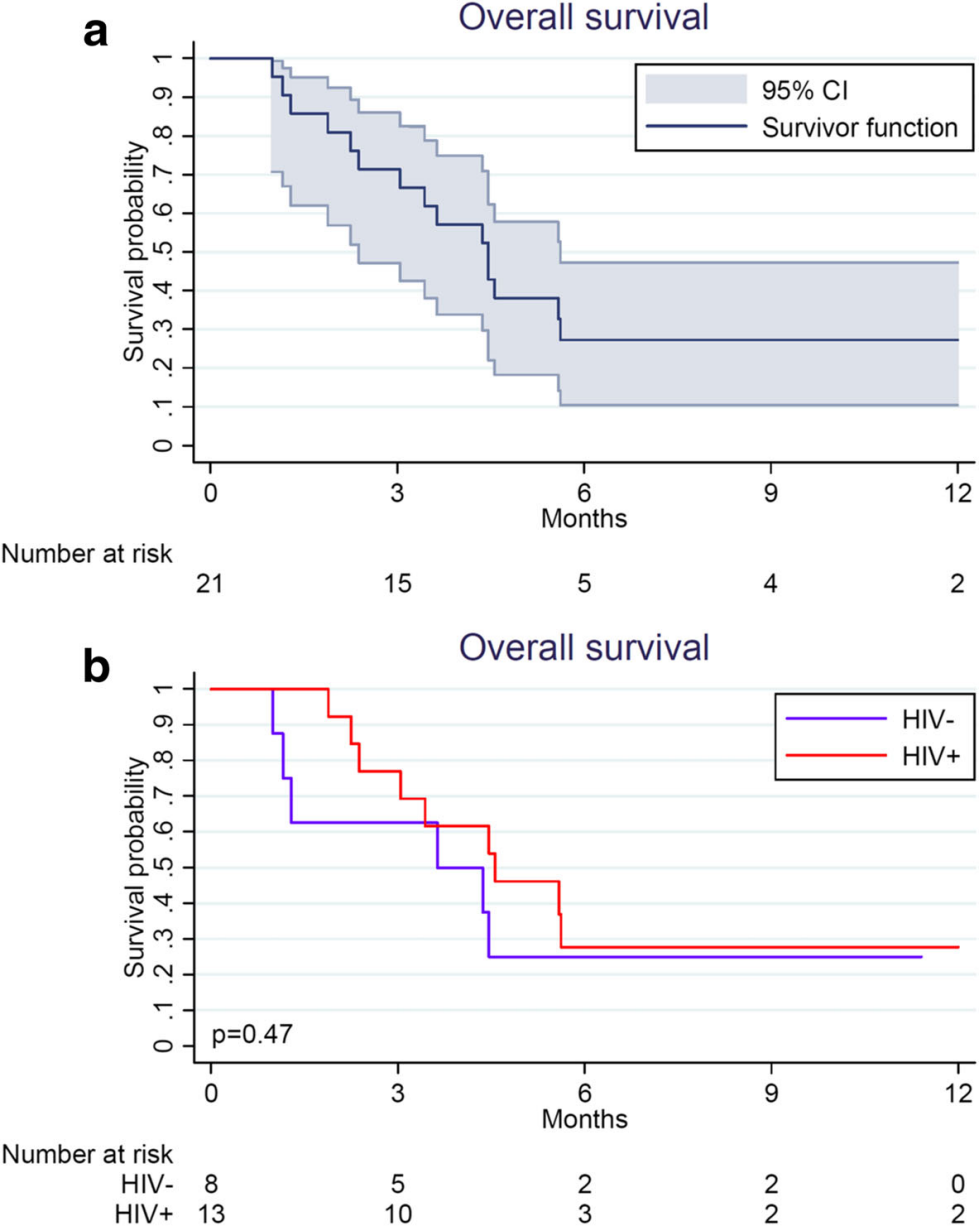
Overall survival for patients with relapsed/refractory lymphoma receiving salvage chemotherapy in Lilongwe. **a** Overall cohort. **b** Stratified by HIV status

Fifteen patients (71%) achieved an objective response to EPIC [3 CR, 12 PR] compared with baseline. Of three patients achieving CR, all had HIV-positive NHL (2 diffuse large B-cell lymphoma, 1 plasmablastic lymphoma) and remained in remission as of August 31, 2016 after 7.2, 14.5, and 15.0 months respectively, including one patient who was able to travel to India for consolidative high-dose therapy with autologous stem cell rescue after achieving CR with EPIC in Malawi. As of August 31, 2016, vital status was known for all 21 patients with no loss to follow-up. Median follow-up time from EPIC initiation was 11.2 months (range 5.1–15.0) among patients still alive. As shown in Fig. 1, median OS 4.5 months [95% confidence interval (CI) 2.4–5.6] for the entire cohort. OS differences were not observed between HIV-infected patients (median 4.6 months, 95% CI 3.0–5.6) and HIV-negative patients (median 4.0 months, 95% CI 1.2–7.6). Of fifteen deaths in the study population, all but two were attributed to progressive lymphoma. Of three patients with relapsed/refractory HL specifically, all were HIV-negative, none achieved CR after EPIC, and two died of progressive HL as of August 31, 2016.

## Discussion

In Lilongwe, we found the modified EPIC regimen to be feasible and well tolerated as salvage treatment for RRL in a severely resource-limited setting in SSA, for patients with and without HIV. Most patients responded, although durability of responses was short with limited OS. Notably, OS was similar to descriptions of salvage chemotherapy for RRL in resource-rich settings, when not followed by consolidative high-dose therapy with autologous stem cell rescue [10].

Our findings highlight an urgent need for better first-line treatment for lymphoma in SSA [2–5]. Education efforts for communities and health care workers can facilitate earlier referral and diagnosis. Supportive care should be standardized and refined, with resource-appropriate incorporation of hematopoietic growth factors. Protocol-guided chemotherapy with defined strategies for monitoring and dose adjustment should be adopted. Ensuring continuous chemotherapy supply and incorporating newer standard-of-care targeted agents is also important, along with greater radiotherapy availability for patients with localized, bulky disease. Finally, strategies for more effectively risk-stratified, response-guided treatment are likely achievable even in the SSA context.

The appropriateness and cost-effectiveness of salvage chemotherapy for RRL in SSA are uncertain. Escalating costs of cancer care pose important ethical considerations and threats to health systems even in resource-rich settings [11, 12]. These issues may be heightened in settings where there are competing needs to maximize public health benefits using limited resources for cancer control, but also to help individual patients as much as possible by applying what is locally available. Low acceptance of palliative care in SSA context has been reported, [13] and in our experience, patients were extensively counseled at the start of salvage chemotherapy about its anticipated limited duration of benefit, but almost uniformly elected to be treated when eligible. Cultural sensitivities may influence patient and provider willingness to forego active treatment, particularly when occasional exceptional responses occur. This is illustrated by three Lilongwe patients who experienced quite long CRs, including one patient achieving second CR with EPIC that allowed subsequent high-dose therapy and autologous stem cell rescue consolidation in India. As cancer programs mature in SSA, developing an appropriate economic and ethical framework to optimally apply available resources will be critical, with strong participation by local stakeholders, and ‘rationing’ treatment beyond first-line may be necessary for those most likely to benefit.

Study strengths include prospective, longitudinal follow-up of RRL cases confirmed using real-time consensus telepathology, supported by IHC and multiple US and Malawi pathologists. Patients underwent detailed and systematic clinical characterization, and those with HIV received concurrent ART in a mature national program. Patients were actively followed with complete outcome ascertainment. We also made efforts to standardize chemotherapy, and our study lacked major exclusions. Limitations include referral bias intrinsic to the Malawi health system, given centralization of cancer services in Lilongwe and Blantyre, the two largest cities. Another limitation is absent death certification in Malawi, leading us to attribute causation through centralized review.

## Conclusion

In summary, salvage chemotherapy using the modified EPIC regimen was feasible and well tolerated as salvage treatment for RRL in Malawi. Overall response rates were high, response durations were short, and OS was limited although a few patients experienced extended remissions. The appropriateness and cost-effectiveness of this approach in severe resource-limited environments in SSA is uncertain, and multifaceted efforts to improve outcomes among newly diagnosed patients in the first line are paramount, to reduce the frequency with which patients require salvage chemotherapy.

## Abbreviations

ABVD: Doxorubicin, bleomycin, vinblastine, dacarbazine
ART: Antiretroviral therapy
CHOP: Cyclophosphamide, doxorubicin, vincristine, prednisone
CR: Complete reponse
EPIC: Etoposide, prednisone, ifosfamide, cisplatin
HIV: Human immunodeficiency virus
HL: Hodgkin lymphoma
ICE: Ifosfamide, carboplatin, etoposide
KCH: Kamuzu Central Hospital
NHL: Non-Hodgkin lymphoma
OS: Overall survival
PR: Partial response
RRL: Relapsed or refractory lymphoma
SSA: Sub-Saharan Africa

## References

[1] Parkin DM, Nambooze S, Wabwire-Mangen F, Wabinga HR (2010). Changing cancer incidence in Kampala, Uganda, 1991-2006. Int J Cancer.

[2] Kingham TP, Alatise OI, Vanderpuye V, Casper C, Abantanga FA, Kamara TB, Olopade OI, Habeebu M, Abdulkareem FB, Denny L (2013). Treatment of cancer in sub-Saharan Africa. Lancet Oncol..

[3] Gopal S, Wood WA, Lee SJ, Shea TC, Naresh KN, Kazembe PN, Casper C, Hesseling PB, Mitsuyasu RT (2012). Meeting the challenge of hematologic malignancies in sub-Saharan Africa. Blood.

[4] Abdel-Wahab M, Bourque JM, Pynda Y, Izewska J, Van der Merwe D, Zubizarreta E, Rosenblatt E (2013). Status of radiotherapy resources in Africa: an International Atomic Energy Agency analysis. Lancet Oncol..

[5] Gopal S, Fedoriw Y, Kaimila B, Montgomery ND, Kasonkanji E, Moses A, Nyasosela R, Mzumara S, Varela C, Chikasema M (2016). CHOP Chemotherapy for Aggressive Non-Hodgkin Lymphoma with and without HIV in the Antiretroviral Therapy Era in Malawi. PLoS One.

[6] Westmoreland KD, Stanley CC, Montgomery ND, Kaimila B, Kasonkanji E, El-Mallawany NK, Wasswa P, Mtete I, Butia M, Itimu S et al. Hodgkin lymphoma, HIV, and Epstein-Barr virus in Malawi: Longitudinal results from the Kamuzu Central Hospital Lymphoma study. Pediatr Blood Cancer. 2017;64(5). doi:10.1002/pbc.26302. Epub 2016 Oct 26. 10.1002/pbc.26302 PMC5529120 27781380

[7] Stanley CC, Westmoreland KD, Heimlich BJ, El-Mallawany NK, Wasswa P, Mtete I, Butia M, Itimu S, Chasela M, Mtunda M (2016). Outcomes for paediatric Burkitt lymphoma treated with anthracycline-based therapy in Malawi. Br J Haematol.

[8] Gopal S, Krysiak R, Liomba NG, Horner MJ, Shores CG, Alide N, Kamiza S, Kampani C, Chimzimu F, Fedoriw Y (2013). Early experience after developing a pathology laboratory in Malawi, with emphasis on cancer diagnoses. PLoS One.

[9] Montgomery ND, Liomba NG, Kampani C, Krysiak R, Stanley CC, Tomoka T, Kamiza S, Dhungel BM, Gopal S, Fedoriw Y (2016). Accurate real-time diagnosis of lymphoproliferative disorders in Malawi through clinicopathologic teleconferences: a model for pathology services in Sub-Saharan Africa. Am J Clin Pathol.

[10] McBride NC, Ward MC, Mills MJ, Eden AG, Hughes A, Cavenagh JD, Lamont A, Newland AC, Kelsey SM (1999). Epic as an effective, low toxicity salvage therapy for patients with poor risk lymphoma prior to beam high dose chemotherapy and peripheral blood progenitor cell transplantation. Leuk Lymphoma.

[11] Young RC (2015). Value-based cancer care. N Engl J Med.

[12] Smith TJ, Hillner BE (2011). Bending the cost curve in cancer care. N Engl J Med.

[13] Harding R, Selman L, Powell RA, Namisango E, Downing J, Merriman A, Ali Z, Gikaara N, Gwyther L, Higginson I (2013). Research into palliative care in sub-Saharan Africa. Lancet Oncol.

